# Determining Factors for the Development of Critical Thinking in Higher Education

**DOI:** 10.3390/jintelligence13060059

**Published:** 2025-05-22

**Authors:** Dora Lucia Jaramillo Gómez, Annie Julieth Álvarez Maestre, Abad Ernesto Parada Trujillo, Carlos Alfredo Pérez Fuentes, Dago Hernando Bedoya Ortiz, Ruth Katherine Sanabria Alarcón

**Affiliations:** 1 PhD Program, Doctorado en Educación y Estudios Sociales, Tecnológico de Antioquia Institución Universitaria, Medellín 050034, Colombia; dora.jaramillo@correo.tdea.edu.co; 2 Psychology Program, Tecnológico de Antioquia Institución Universitaria, Medellín 050034, Colombia; 3 Social Work Program, Tecnológico de Antioquia Institución Universitaria, Medellín 050034, Colombia; abad.parada@tdea.edu.co; 4 Facultad de Derecho y Ciencias Forenses, Tecnológico de Antioquia Institución Universitaria, Medellín 050034, Colombia; carlos.perez77@tdea.edu.co; 5 Facultad de Ingeniería, Tecnológico de Antioquia Institución Universitaria, Medellín 050034, Colombia; dago.bedoya@tdea.edu.co; 6 Health Sciences, UCAM-Universidad Católica San Antonio de Murcia, 30107 Murcia, Spain; rksanabria@alu.ucam.edu

**Keywords:** critical thinking, higher education, university

## Abstract

This study arises from the growing need to train professionals capable of confronting and analyzing the overabundance of information in an increasingly complex world, where critical thinking is seen as an indispensable skill for informed decision making and problem solving. To this end, a systematic narrative review methodology was applied to the scientific literature, compiling data from various international databases. The results reveal that physiological factors (memory, attention, nutrition and physical activity), psychological factors (cognitive biases, fear of ambiguity, and metacognition), sociocultural factors (diversity, inequality, and cultural norms), technological factors (digitalization, use of AI, and digital literacy), and educational factors (active pedagogical strategies and collaborative work) play a determining role in the development of critical thinking in higher education. The discussion emphasizes the complex interaction between these factors and underscores the need for holistic approaches that strengthen both cognitive competencies and emotional well-being. In conclusion, we recommend designing comprehensive training interventions that consider the identified factors, promoting inclusive and reflective environments, aimed at developing critical, autonomous graduates capable of facing contemporary challenges.

## 1. Introduction

Critical thinking has established itself as an essential component of higher education, particularly in contexts where the overabundance of information demands skills of discernment and rigorous assessment (Aston 2024; Gonzalez-Mohino et al. 2023; Sutherland and Incera 2021). Various authors agree that this competency involves the ability to analyze, evaluate, and synthesize information, questioning assumptions and contrasting multiple perspectives in order to make informed judgments (Ennis 1991; Facione 2011; Halpern and Dunn 2021; Saiz and Rivas 2023; Thornhill-Miller et al. 2023).

Despite its recognized importance, the scientific literature highlights multiple challenges to its development. These include the predominance of traditional pedagogical methodologies, the lack of attention paid to metacognitive skills, and gaps in teacher training, as factors that limit students’ reflective potential (Alsaleh 2020; Dissen 2023; Pupkis and Saulius 2023; Valavičienė et al. 2020). Furthermore, cultural differences and variations in available technological resources can significantly influence students’ ability to question assumptions and deeply analyze the information they receive (Crone and van Goch 2024; Jasmadi et al. 2024; Kerruish 2025). Similarly, recent studies point to the importance of motivation, autonomy, and a willingness to collaborate as key elements that can enhance or, conversely, hinder the acquisition of critical skills (Arhip 2022; Campo et al. 2023; Guamanga et al. 2025).

In parallel, the increasing digitalization of higher education institutions has highlighted the need to integrate technological tools not only as a means of accessing information but also as a means of developing reflective thinking processes, since their instrumental use can lead to superficial learning practices (Song and Cai 2024; Van den Berg and du Plessis 2023; Wang 2024). Meanwhile, research on pedagogical strategies confirms the importance of participatory and collaborative methodologies, such as problem-based learning or project-based learning, to encourage the confrontation of ideas and the evaluation of arguments, which are intrinsic dimensions of critical thinking (Abujreiban 2023; Bhuttah et al. 2024; Van et al. 2022). However, these initiatives require an institutional environment that values inquiry and debate, as well as teachers prepared to guide and provide feedback on complex reasoning processes (Kazemian et al. 2021; Koseni et al. 2025; Nuritdinov 2024; Tsopra et al. 2023).

Along these lines, the measurement of critical thinking is also a source of debate for determining a skill’s level of development. Recent research shows that scores on the most commonly used tests share a substantial portion of variance with general cognitive abilities, especially fluid intelligence described in the tri-stratum model (Carroll 1993, 2005; Thornhill-Miller et al. 2023). Furthermore, existing instruments are based on heterogeneous theoretical frameworks, reasoning skills, dispositions, and problem solving, which lead to poorly comparable results and limit the accumulation of convergent evidence. It has also been argued that many items rely on declarative knowledge or vocabulary, introducing cultural and linguistic variance that contaminates the scores obtained (Liu et al. 2024). These moderate-high correlations persist even when controlling for executive functions, suggesting partial redundancy between the constructs of CT and general cognitive ability (Halpern and Dunn 2021). These findings underscore the need for more rigorous assessment strategies that isolate the unique contribution of CP and strengthen its convergent and discriminant validity in future studies (Rane-Szostak and Robertson 1996).

Based on this problem, the objective of this study was to analyze the factors that positively or negatively influence the development of critical thinking in higher education students, considering both individual variables—motivation, metacognitive skills, and cognitive autonomy—and contextual elements, such as teaching practices, institutional culture, and technological resources, that shape the educational environment. The aim is to provide a framework that guides the essential elements in the design of more effective interventions and training programs for critical thinking, promoting the development of graduates with the ability to reason reflectively in increasingly complex contexts.

## 2. Materials and Methods

**Figure 1:**
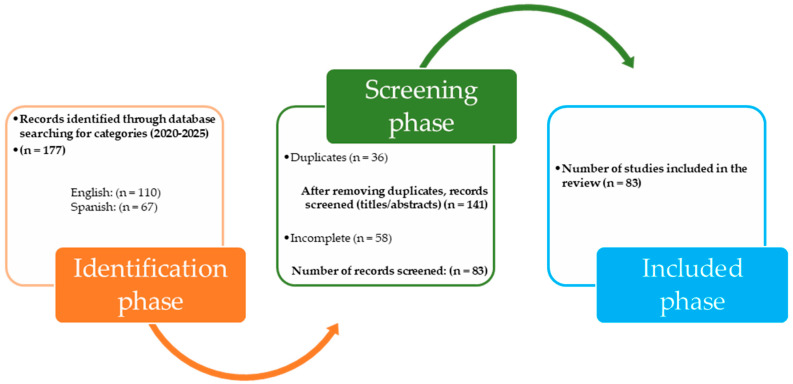
Flowchart for research selection. During the identification phase, international databases were consulted, allowing us to retrieve 177 records corresponding to studies published between 2020–2025. Of these, 110 were written in English (62%) and 67 in Spanish (38%). In the screening phase, automatic and manual filters were applied. First, 36 duplicate records (20.3%) were eliminated, which refers to studies repeated in the different databases, obtaining 141 unique references. Titles, abstracts, and article content were then reviewed; in this stage, 58 incomplete records were discarded (corresponding to no access to the full text or with insufficient methodological data), equivalent to 41.1% of the articles evaluated. Finally, 83 studies met the minimum quality criteria and were entered into the data matrix, forming the basis for the eligibility and qualitative synthesis phases.

This research conducted a systematic, narrative review of scientific literature (Guirao-Goris 2015; Sánchez-Meca 2022). The phases developed the research question, the determination of the criteria to be investigated, bibliographical search in indexed journals, screening, and finally, the analysis and writing of the subject in question (Onwuegbuzie and Frels 2016). The objective of the study was to analyze the factors that positively or negatively influence the development of critical thinking in higher education students. This information will allow for the optimization of educational processes by implementing strategies or programs that efficiently promote critical thinking (Figure 1).

While this review focuses on the factors that determine the development of critical thinking (CT), it recognizes the existence of a larger body of work devoted to other generic cognitive skills, for example, academic communication, decision making, and creativity, which have also been the subject of recent syntheses (Tuononen et al. 2022). The literature compares these competencies under the umbrella of “generic skills”, emphasizing that each has specific training trajectories and pedagogical strategies (Goriot et al. 2024). In contrast, this analysis delimits CT as a central construct because (a) it exhibits a consensus on its cross-cutting relevance, and (b) it presents its own methodological controversies, especially regarding measurement, which require a focused approach.

However, to distinguish this review from larger studies, two criteria were adopted. First, we included only research that explicitly analyzes CT and reports its links with physiological, psychological, sociocultural, technological, and educational variables, excluding works that address generic skills in an aggregated manner. Second, we contrasted the findings with contemporary models of transversal competency development, which made it possible to identify points of convergence, for example, the role of cognitive self-regulation and areas where CT displays differential patterns, such as its dependence on formal argumentation tasks and evidence evaluation (Babashamasi et al. 2022; Rivas et al. 2022). This provides a specialized synthesis that, while engaging with the broader skills agenda in higher education, delves into the methodological and conceptual challenges inherent to critical thinking.

### 2.1. Phase 1 Study Criteria

- Research question: What are the factors that influence the development of critical thinking in higher education students?
- Design: Qualitative, quantitative or mixed studies were collected.
- Categories: studies on physiological, cognitive, psychological, athletic, educational, emotional, technological, social, and cultural factors that influence the development of critical thinking in higher education.
- Search fields used: all text (all text), title (title), subject headings (subject terms). First, the exact search was used with quotation marks. Subsequently, the Boolean “and” and “or” were used to refine the search. The Boolean “not” was also used to exclude unrelated or contextual searches.
- Participants: higher education students.
- Language: studies written in Spanish and English.
- Time range: studies carried out between 2020–2025.
- Exclusion: Incomplete documents (without access to the full text or with insufficient methodological data) or those applied to child, adolescent, or disabled student populations were not taken into consideration.
- Electronic databases: Proquest, Cengage, Scopus, EBSCOhost, Redalyc, Dialnet, Cengage Learning, ERIC, Springer, WOS, Elsevier, JURN, Teseo, World Wide Science, ASSIA, Google Scholar, Doaj, Gale Powers, and APA PsycInfo.

### 2.2. Phase 2 Instruments

Data logging. The information was structured in an Excel database, linking the following categories in a matrix: year, source, location, language, reference, study title, design, study category, associated subcategory, abstract, problem, objective, theoretical references, type of methodology, methodology, design, results, and conclusions. Consequently, a flowchart based on the prism method was constructed, synthesizing the collected information.

### 2.3. Phase 3 Data Analysis

In this final stage of the methodological design, content analysis was used. Thus, the main analysis process was qualitative coding through the identification of units of meaning, the creation of categories, and their classification (Sánchez-Meca 2022).

## 3. Results

The results of the quantitative synthesis of the 83 studies included are presented below. Overall, the quantitative synthesis of the literature reveals an uneven landscape in the attention that different studies pay to each determining dimension of critical thinking in higher education. See Table 1. Of the total of 83 studies in the sample, 28 (33.7%) focus on psychological factors, confirming the priority that current research gives to processes such as metacognition, motivation, and attentional control. These are followed in magnitude by works that address educational variables (18 studies; 21.7%) and technological variables (17 studies; 20.5%), indicating that the quality of teaching methodologies and digital mediation are considered key levers for strengthening critical thinking at the university level. Sociocultural factors, represented by 13 studies (15.7%), appear in the background, highlighting the influence of access to resources, cultural diversity, and the participatory dynamics of the environment. Finally, only seven studies (8.4%) explore physiological variables, such as sleep, nutrition or physical activity, which reveals a still nascent area and suggests the need to more strongly integrate the dimension of physical well-being in future explanatory models.

For a detailed view of the findings, five separate tables are included below (Table 2, Table 3, Table 4, Table 5 and Table 6); each one compiles studies corresponding to one of the identified factors (physiological, psychological, sociocultural, technological, and educational), specifying the author, year, and title of the study.

### Key Observations

Predominance of internal factors: More than a third of the evidence focuses on psychological variables, reflecting the centrality assigned to cognitive-affective processes (metacognition, motivation, attentional control) in explaining critical thinking (CT).

Importance of the pedagogical dimension: Educational and technological factors account for 42% of the studies, highlighting that the design of learning experiences and digital mediation are strategic levers for enhancing CT in universities.

Less attention paid to physiological factors: Only 8% of the studies address basic variables, such as sleep, nutrition, or physical activity, despite growing evidence that they influence cognitive self-regulation; a niche for future research is identified here.

Ecosystemic view: The presence of sociocultural factors (15.7%) indicates that the context—access to resources, diversity, and democratic culture—modulates the effectiveness of pedagogical and technological interventions, suggesting the need for integrated approaches.

Thematic balance with respect to the total number of studies reviewed (*n* = 83): the percentage distribution confirms that, although research interest is relatively balanced between educational/technological and psychological dimensions, there are underexplored areas (physiological) that could enrich holistic models on the development of CT.

The study’s results reveal a complex interrelationship between multiple factors that, together, determine the development of critical thinking in higher education. First, physiological factors, such as sleep quality, nutrition, and physical activity, are shown to be fundamental for sustaining information processing and retention, establishing the cognitive foundation necessary to address complex problems. Psychological factors are also found, showing that the presence of cognitive biases and fear of ambiguity can limit analytical capacity, while metacognition acts as a tool to counteract these limitations, promoting a more objective evaluation of information.

On the other hand, sociocultural factors highlight the importance of the context in which students develop, since diversity, inequalities, and cultural norms shape environments that can either foster or restrict the development of critical skills. Similarly, technological advances, measured through digitalization, the use of artificial intelligence, and digital literacy, offer new opportunities and challenges, depending on their integration into the educational process. Thus, educational factors, evidenced in the implementation of active pedagogical strategies and the promotion of collaborative work, are positioned as key elements for transforming the interaction of all these factors into meaningful learning, capable of producing critical, autonomous graduates prepared to face contemporary challenges.

Therefore, the relevance of the findings lies in the confirmation that the development of critical thinking is a multifactorial process in which each dimension—physiological, psychological, sociocultural, technological, and educational—contributes in an indispensable way to the comprehensive development of the student. This holistic approach not only allows for the identification of specific areas of intervention but also guides the design of innovative pedagogical strategies that strengthen both cognitive competencies and emotional well-being, making it crucial for the preparation of professionals capable of navigating and transforming complex environments.

## 4. Discussion

The systematic review conducted identified key factors that influence the development of critical thinking in higher education. The following section addresses the most relevant findings. This provides a clearer view of how different variables can enhance or hinder students’ ability to rigorously reflect on and evaluate information, laying the foundation for a more comprehensive understanding of this educational process.

### 4.1. Physiological Factors

**Figure 2:**
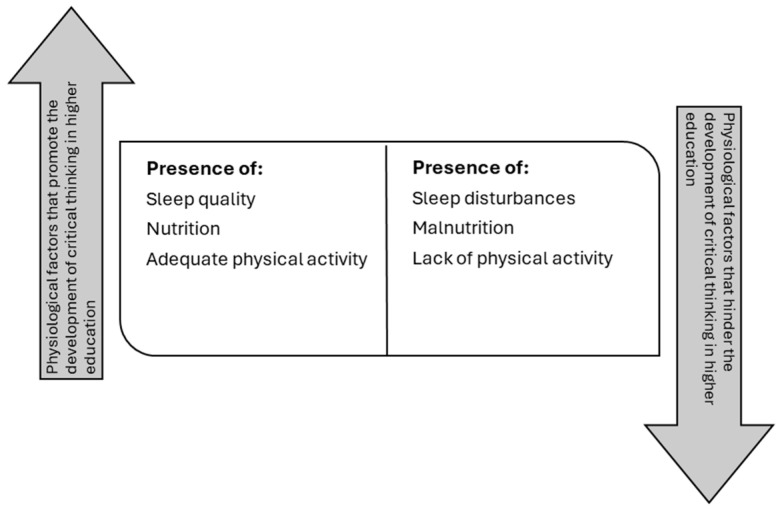
Physiological factors that determine the development of critical thinking in higher education students.

The development of critical thinking in higher education students is profoundly influenced by a series of interrelated physiological factors, including sleep quality, nutrition, and physical activity. These elements form a complex network that directly impacts the cognitive abilities necessary to thoughtfully address complex problems and make informed decisions (Figure 2).

Sleep disturbances, excess, restriction, and, even more crucially, poor quality of sleep, impair key cognitive processes for critical thinking among college students by decreasing sustained attention, weakening working memory, and dysregulating emotional responses (Pereira et al. 2024; Sidra Ilyas et al. 2024). This triple disruption generates a cycle in which daytime sleepiness and fatigue increase mental workload, elevate stress, and induce low-grade systemic inflammation, thus deepening difficulties in decision making and diminishing academic performance. Therefore, safeguarding both the duration and quality of rest is emerging as a decisive factor in preserving cognitive resilience and sustaining higher-order reasoning in higher education (Mehta 2022; Pereira et al. 2024).

On the other hand, a proper and balanced diet, especially rich in omega-3 fatty acids and antioxidants, strengthens basic cognitive functions, such as memory, concentration, and problem solving, thus promoting analytical and critical thinking skills (López-Ruiz et al. 2021; Wassenaar et al. 2020). Nutritional deficiencies can reduce cognitive capacity, affecting mental clarity and limiting the ability to perform critical analysis (Suárez et al. 2020). Another factor is regular physical activity, which improves the brain’s executive functions, such as inhibitory control and cognitive flexibility, which are essential elements for effective critical thinking (Ekerer et al. 2024; Suárez et al. 2020).

In this regard, the design of effective pedagogical strategies must comprehensively consider these physiological factors, not only to enhance academic skills but also to foster emotional and cognitive well-being, which is essential for developing individuals capable of successfully facing critical and complex challenges in the future. Additionally, the authors of this study consider it relevant to mention that the search strategy also included terms related to alcohol and drug use, hydration status, chronic diseases, and genetic indicators. However, no studies within the last 5 years were located that evaluated their specific impact on critical thinking in higher education students, so they did not meet the inclusion criteria. This reveals an empirical gap, where the absence of research directly linking these factors with the development of critical thinking constitutes a priority for future studies.

### 4.2. Psychological Factors

**Figure 3:**
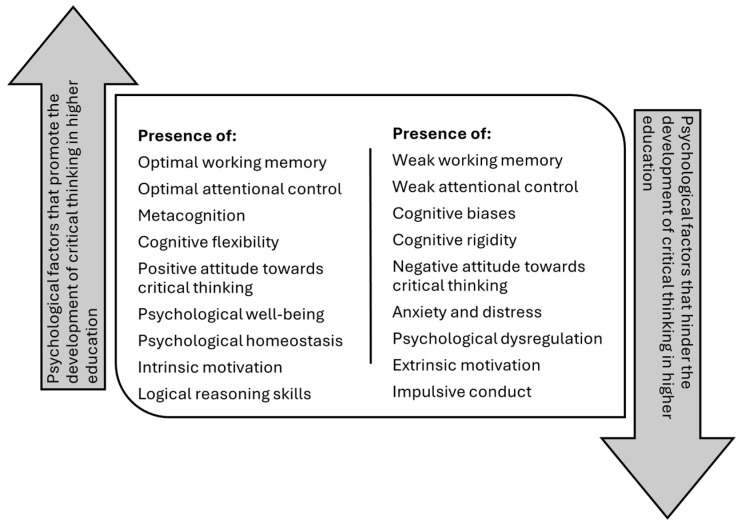
Psychological factors that determine the development of critical thinking in higher education students.

Through the studies analyzed, it was possible to infer that the development of critical thinking is conditioned by various psychological factors (Aston 2024; Guamanga et al. 2025; Kerruish 2025) (Figure 3). First, attention control allows us to focus on relevant information and filter out distractions, increasing the effectiveness of critical analysis (Robison and Garner 2024). Although the relationship between attention and critical thinking can be complex, the ability to maintain focus is crucial for developing deep understanding and performing rigorous evaluations of information (Rivas et al. 2022; Robison and Brewer 2022).

In this sense, the practice of sustained attention strengthens the connection with academic content, increasing students’ reflective and analytical capacities when faced with complex problems, thus facilitating more informed decision making (Arhip 2022). Additionally, working memory facilitates the efficient retention and manipulation of information during complex cognitive tasks, allowing for the simultaneous integration of multiple knowledge elements, which is essential for critical problem solving (Zhao and Vogel 2025).

Additionally, a series of recent studies highlights the impact of cognitive biases, including confirmation bias, which leads students to prioritize information consistent with their prior beliefs, thus limiting their capacity for objective and balanced analysis. This inclination toward pre-existing confirmation not only affects the objectivity of judgment but also hinders the critical evaluation of contradictory evidence, a crucial aspect in advanced academic contexts (Amin et al. 2024; Smith and Peloghitis 2020).

Closely related to the above, the fear of ambiguity is another significant psychological factor identified by various researchers. Students from educational environments predominantly focused on certain and concrete answers have a lower tolerance for ambiguous situations, which are inherent to critical thinking activities (Aston 2024; Saiz and Rivas 2023). This discomfort with uncertainty can generate psychological resistance to academic tasks that demand complex analysis and tolerance for contradiction or ambivalence (Crone and van Goch 2024).

On the other hand, framing effects, how information is presented or formulated, play a central role in the activation of critical thinking processes. It has been observed that different modes of presentation can distort or favor students’ rational evaluation, impacting their academic decisions and analytical skills (Aston 2024; Rarita 2023). This phenomenon indicates that the quality of critical thinking depends not only on individual competencies but also on contextual factors that regulate the interpretation and processing of information (Kerruish 2025).

In parallel, metacognition consistently emerges as an essential determinant in promoting critical thinking. Recent studies underline that the conscious application of metacognitive strategies, such as continuous self-assessment and reflective questioning, facilitates better monitoring and regulation of cognitive processes in university students (Garcés-Suarez 2024; Guamanga et al. 2025; Kazemian et al. 2021; Rivas et al. 2022). Furthermore, pedagogical methods, such as problem-based learning (PBL) and structured discussions, reinforce metacognitive capacity, allowing students to identify gaps in their understanding, systematically evaluate evidence, and actively refine their reasoning processes (Huamán 2022; Lugo et al. 2022).

The influence of personal attitudes and beliefs toward critical thinking has also been widely highlighted in contemporary research. In particular, a willingness to engage in cognitive reflection and a value for evidence-based reasoning are significantly correlated with improved critical thinking skills and higher academic achievement (Van et al. 2022; Simonovic et al. 2022). Educational interventions aimed at promoting positive attitudes toward critical thinking, especially those focused on how to think rather than what to think, demonstrate effective results in terms of developing analytical skills, tolerance for ambiguity, and cognitive flexibility (Berberena and Wirzberger 2021; Antonova et al. 2021; Villarruel-Diaz and Portocarrero-Gutiérrez 2021).

From an emotional dimension, anxiety emerges as a relevant physiological factor, since high levels of generalized anxiety can significantly reduce academic self-efficacy and hinder the development of critical thinking by limiting deep and reflective participation in complex learning processes (Abanto-Ramirez et al. 2024; Wang 2024). Excessive worry in higher education students can hinder critical thinking capacity by generating a constant state of alert that overloads working memory and reduces cognitive flexibility (Huamán-Tapia et al. 2023). This emotional overstimulation makes it difficult to objectively evaluate information and formulate reasoned judgments, since students tend to focus on potential threats rather than analyzing data in a reflective and systematic manner (Jia 2024).

Likewise, psychological well-being is emerging as an increasingly relevant factor in the context of critical thinking. Recent research shows that dimensions of well-being, such as self-acceptance, control over one’s environment, and a clearly defined purpose, correlate positively with a more open and resilient disposition toward critical thinking (Guamanga et al. 2025; Hyseni Duraku et al. 2024; Wang 2024). Intrinsic motivation is also key, as it fosters active and reflective learning, increasing students’ willingness to confront deep and complex cognitive challenges (Chemagosi 2024; Huaire-Inacio et al. 2023). In contrast, sustained emotional stress and a lack of motivation negatively affect the depth and quality of the intellectual engagement required to critically analyze complex concepts and problems (Padmanabhan 2024; Wang 2024).

Additionally, mastery of logic and problem-solving tasks completes the picture of key psychological factors that foster critical thinking in the university setting. Specific training in logic increases students’ analytical skills, allowing them to effectively distinguish valid from invalid arguments and adequately evaluate the evidence presented (Li 2024; Pedorych 2024). Frequent participation in tasks that pose unconventional challenges fosters the ability to differentiate relevant from irrelevant information, thereby strengthening the skills associated with critical thinking (Van Damme et al. 2023).

### 4.3. Sociocultural Factors

**Figure 4:**
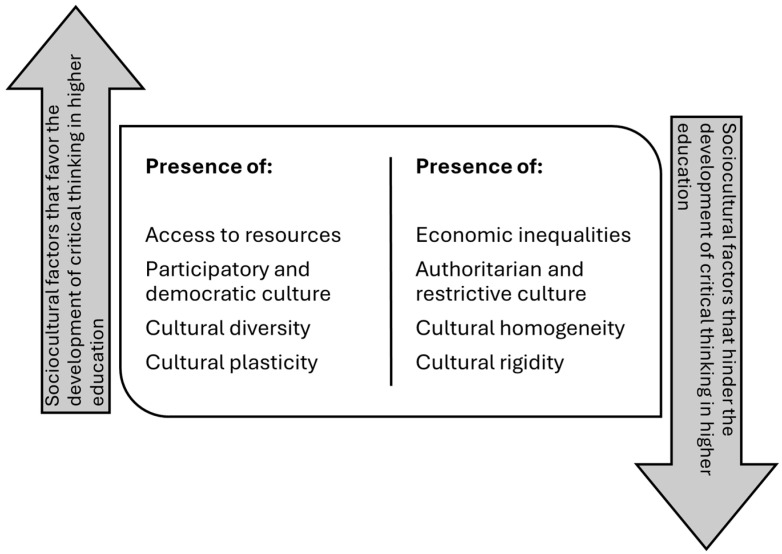
Sociocultural factors that determine the development of critical thinking in higher education students.

The interaction between the social environment, the educational context, and cultural diversity creates a complex framework in which critical thinking is not only encouraged but also conditioned by prevailing structures (Afzal et al. 2023; Darcie et al. 2024) (Figure 4). While formal education is an essential vehicle for its development, the influence of teaching systems, institutional regulations, and pedagogical practices cannot be understood in isolation, as they depend on how society values questioning, analysis, and intellectual autonomy (Nuritdinov 2024; Van Damme et al. 2023).

A central aspect that emerges from the studies is the relationship between socioeconomic status and opportunities to develop critical thinking (Kerruish 2025). The availability of resources, access to trained teachers, and the opportunity to participate in enriching educational experiences create a scenario where students from advantaged backgrounds tend to show a greater willingness to engage in argumentation and critical analysis (Aboukhousa et al. 2024; Shukla and Kumari 2024). However, this relationship is not deterministic, as other factors, such as the learning culture in the classroom, can counteract or enhance these conditions. An educational environment that encourages active participation and uncertainty-based learning can compensate for economic inequalities and provide spaces where students develop more complex reasoning strategies (Darcie et al. 2024; Liu and Pásztor 2023).

In this sense, cultural diversity in the classroom emerges as an ambivalent factor. On the one hand, exposure to multiple perspectives enriches students’ ability to evaluate different points of view and formulate stronger arguments (Singla et al. 2024; Mejía-Manzano et al. 2022). However, diversity also introduces challenges, especially when cultural differences generate communication barriers or when pedagogical strategies are not designed to equitably integrate all the voices present in the classroom. In contexts where homogenization of discourses prevails, the capacity for critical thinking can be restricted, limiting the transformative potential that cultural diversity could bring to higher education (Afzal et al. 2023; Escobar Domínguez 2024).

Another relevant aspect is the relationship between critical thinking and the cultural norms that regulate social interaction. In societies where questioning authority is discouraged or where education prioritizes memorization over reflection, students may encounter obstacles in developing advanced analytical skills (Mejía-Manzano et al. 2022; Novoa Seminario and Sandoval Rosas 2023). The tension between the hierarchical structure of certain educational models and the need to foster critical thinking is evident in disciplines such as law, where educational systems that incorporate argumentation and critical analysis into the curriculum offer a more conducive framework for its development. However, when cultural norms favor obedience and passive learning, the possibilities of fostering a critical mindset are significantly limited (Nuritdinov 2024).

Consequently, the interaction between economic, cultural, and educational factors demonstrates that critical thinking does not develop in a vacuum, but rather in a complex ecosystem where higher education plays a crucial, but not exclusive, role. Educational systems that promote flexible learning environments, active methodologies, and an argument-based approach can counteract certain limitations imposed by socioeconomic or cultural factors. However, when education is treated as a commodified product or when power structures limit the ability to question and generate new ideas, the possibilities of consolidating critical thinking are restricted. In this context, it is imperative that pedagogical strategies not only address the teaching of critical thinking as a set of isolated skills, but also consider the social conditions that influence its development (Koseni et al. 2025; Walker and LeBoeuf 2022).

### 4.4. Technological Factors

**Figure 5:**
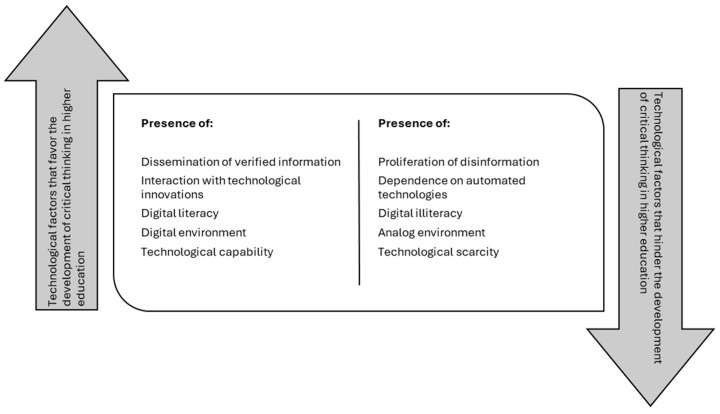
Technological factors that determine the development of critical thinking in higher education students.

Digital tools have been shown to enhance students’ analytical and reflective abilities by providing access to diverse sources of information and facilitating interaction with multiple perspectives (Figure 5). The role of digitalization in educational processes introduces a novel dimension to the analysis of critical thinking (Vélez et al. 2024). The growing reliance on technological tools can expand opportunities for accessing knowledge and strengthening certain cognitive abilities, but it can also generate dynamics of information dependence that reduce students’ ability to analyze independently (Al-Zou’bi 2021; Fitriani and Prodjosantoso 2024).

In this context, this positive impact is not without challenges, as overexposure to unverified information and dependence on automated technologies can hinder the capacity for independent reasoning and informed decision making (Foote and McMorran 2024; Gonzalez-Mohino et al. 2023). Furthermore, interaction with memory technologies modifies reasoning processes, which can enhance or restrict the development of critical thinking, depending on the use made of these resources (Kerruish 2025). In this sense, the digitalization of education should not be interpreted solely as a step forward, but as a phenomenon that redefines the conditions under which students exercise their critical thinking.

The use of technological innovations in higher education has shown an improvement in the effectiveness of critical thinking, increasing students’ professional preparation and strengthening their ability to critically evaluate information (Pedraja-Rejas et al. 2024). However, massive access to digital information also generates tensions, since the proliferation of misinformation can hinder the formation of informed judgments and encourage uncritical consumption of content (Foote and McMorran 2024; Lopez-González et al. 2023; Wang 2024). In this context, digital literacy becomes a key factor, since its adequate implementation not only mitigates the risks associated with misinformation but also optimizes academic performance by allowing a more reflective use of digital resources (Al-Zou’bi 2021; Mango-Quispe et al. 2024).

The impact of artificial intelligence (AI) on critical thinking is mixed. On the one hand, AI tools can improve data analysis, problem solving, and decision making by automating complex processes and providing immediate feedback (Van den Berg and du Plessis 2023; Vera 2023). However, overreliance on these systems can reduce active engagement in learning, leading to a decrease in students’ willingness to question and reflect autonomously. Furthermore, the reliability and accuracy of AI models raise ethical and epistemological concerns that impact the development of critical skills, as students may internalize information without a rigorous assessment process (Jasmadi et al. 2024; Nguyen et al. 2024).

Likewise, digital learning platforms have shown a varied impact on the development of critical thinking. While they facilitate access to interactive educational materials and foster student engagement, their influence on academic performance is not directly related to an improvement in critical thinking skills. This suggests that the use of technology in the classroom, without an appropriate pedagogical approach, may not be sufficient to substantially enhance critical thinking (Bhuttah et al. 2024; Ravikumar et al. 2025). In this sense, the integration of mobile applications, educational games, and e-learning platforms can promote collaborative environments that stimulate critical thinking, provided there is an orientation that encourages reflection and intellectual autonomy in students (Fitriani and Prodjosantoso 2024).

Based on the above, the impact of digitalization on higher education highlights the need for pedagogical strategies that balance access to technology with the strengthening of metacognitive skills. While the appropriate use of digital tools can enrich teaching and civic participation by developing analytical and evaluation skills, their application without adequate pedagogical mediation can lead to technological dependence that weakens students’ critical thinking skills (Al-Zou’bi 2021). In this way, technology is not an end in itself, but a means that, when used strategically, can transform education and strengthen the development of critical citizens in a digitalized society (Foote and McMorran 2024; Gonzalez-Mohino et al. 2023).

### 4.5. Educational Factors

**Figure 6:**
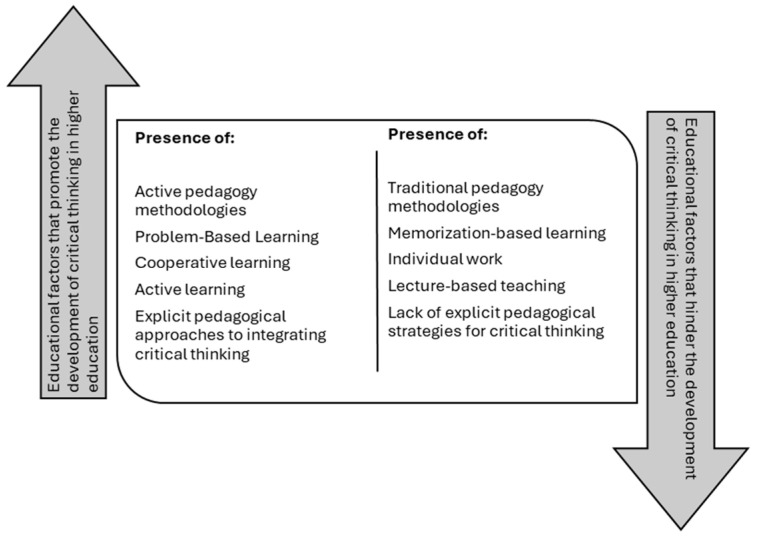
Educational factors that determine the development of critical thinking in higher education students.

The development of critical thinking (CT) in higher education students has been the subject of detailed analysis in various research studies, which point to the essential role of active pedagogy methodologies in the development of critical skills (Dissen 2023; Song and Cai 2024) (Figure 6). These investigations agree that the use of strategies, such as problem-based learning (PBL), case studies, and inquiry-based learning (IBL), is essential to foster CT, since these methodologies allow students to face complex situations that require in-depth analysis, reasoning and argumentation, thus favoring the development of advanced cognitive skills (Campo et al. 2023; Villarruel-Diaz and Portocarrero-Gutiérrez 2021). Furthermore, these strategies promote active student participation in the learning process, allowing them to question, reflect and construct their own arguments, which is essential for critical thinking (Marín Ube et al. 2025; Pincay Parrales 2022; Sánchez Gonzales and Nagamine Miyashiro 2021; Song and Cai 2024; Villarruel-Diaz and Portocarrero-Gutiérrez 2021).

A constant in the reviewed studies is the importance of collaborative work as a key factor for the development of CT. Interaction between peers in debate and discussion spaces not only encourages deep reflection but also enriches students’ ability to question and analyze different perspectives on the same topic, a process that is closely linked to the improvement of thinking (Alsaleh 2020; Marín Ube et al. 2025). Furthermore, the use of pedagogical techniques such as teamwork, debate, and joint reflection allows students to develop argumentation, analysis, and problem-solving skills, skills that are fundamental components of CT (Abujreiban 2023; Mora-Ramírez 2023; Sánchez Gonzales and Nagamine Miyashiro 2021).

A prominent aspect of several studies is the influence of teaching methodology in fostering critical thinking. The predominant use of traditional teaching methods, based on lectures, limits students’ opportunities to practice and develop critical thinking skills (Huaire-Inacio et al. 2023; Villarruel-Diaz and Portocarrero-Gutiérrez 2021). In contrast, methods that encourage active participation and reflection, such as the use of paradoxes or the promotion of debate, prove to be more effective in stimulating problem-solving skills and informed decision making (Pincay Parrales 2022; Sánchez Gonzales and Nagamine Miyashiro 2021). Research indicates that teachers, although aware of the importance of critical thinking, must rethink their teaching strategies to provide spaces where students can question, analyze, and reflect more deeply on the topics covered (Mora-Ramírez 2023; Sutherland and Incera 2021).

The use of digital technologies in the classroom is another factor that has been shown to have a positive impact on the development of critical thinking. The incorporation of interactive tools and digital platforms fosters more flexible and dynamic learning, allowing students to access information, reflect on it, and make decisions based on critical analysis (Cumandá et al. 2022). Interactive learning not only improves students’ digital competence but also enhances their ability to critically evaluate and reflect on different sources of information. In this way, technologies act as a catalyst for the development of CT by offering new forms of interaction and reflection (Song and Cai 2024).

Furthermore, the use of recreational activities has emerged as a crucial factor in fostering critical thinking. Various studies have shown that game dynamics, such as role-playing games or group problem-solving activities, are extremely effective in developing logical reasoning, decision-making, and critical analysis skills (Moya Gómez 2024). These recreational activities not only encourage creativity and free thinking but also promote active participation and dynamic learning, essential elements for the development of CT. The interaction and analysis that occur in the recreational context facilitate the construction of new knowledge and the development of advanced cognitive skills (Garces et al. 2024; Thornhill-Miller et al. 2023).

The use of explicit pedagogical approaches to integrate critical thinking into the curriculum has proven to be an important factor in its development. An explicit approach to critical thinking involves intentional and systematic teaching of critical skills, in which students are guided through structured processes of reflection, analysis, and argumentation. This type of teaching not only improves academic performance but also strengthens students’ cognitive skills and critical dispositions. Research has found that an explicit focus on critical thinking significantly improves students’ skills, helping them develop fundamental competencies for addressing complex problems in diverse areas of knowledge (Dissen 2023; Jones et al. 2023).

## 5. Conclusions

The study shows that the development of critical thinking in higher education students is a multifactorial process, in which physiological, psychological, sociocultural, technological, and educational dimensions converge interdependently. The ability to process complex information and make informed decisions is based on elements such as working memory and attention control, which allow for the retention and manipulation of relevant data. However, these cognitive functions are modulated by emotional and physical factors, such as adequate nutrition, physical activity, and the practice of sustained attention, which together strengthen academic performance. Furthermore, high levels of anxiety can diminish self-efficacy, hindering the reflective approach to complex problems and generating a negative cycle in the disposition for critical analysis.

Similarly, psychological determinants, including cognitive biases and fear of ambiguity, shape students’ predisposition toward self-assessment and metacognitive regulation. The conscious application of metacognitive strategies is essential to counteract these biases, allowing for more objective reflection and critical evaluation of information, which translates into better academic performance. In parallel, sociocultural factors, such as socioeconomic inequalities, cultural diversity, and applied educational practices, generate contexts that can enhance or limit the development of critical skills, underscoring the importance of promoting inclusive and participatory environments.

Digitalization and the use of technologies in education emerge as a dual component, offering valuable resources for accessing information and collaborative interaction, but also posing challenges related to information dependence and the spread of misinformation. Digital literacy and adequate pedagogical mediation are essential to guide students toward reflective and autonomous use of technological tools. Similarly, the implementation of innovative pedagogical methodologies, such as problem-based learning, case studies, and collaborative work, allows not only for the strengthening of advanced cognitive skills but also for the explicit integration of critical thinking into the curriculum.

In conclusion, the research highlights the need to adopt a holistic approach to the design of educational programs or strategies, one that harmoniously and synergistically integrates the various factors that influence the development of critical thinking. It is recommended to develop programs that, in addition to enhancing cognitive skills, address emotional well-being and strengthen students’ metacognitive capacity, thereby developing critical, autonomous, and resilient individuals capable of analyzing, questioning, and transforming their environment in an informed and reflective manner.

## Figures and Tables

**Table 1:** Results and emerging categories of the selected studies.

Determining factors for the development of critical thinking in higher education	Physiological factors Number of studies: (*n* = 7)	Sleep quality
		Nutrition
		Physical activity
	Psychological factors Number of studies: (*n* = 28)	Attentional control
		Working memory
		Metacognition
		Cognitive flexibility
		Positive attitude towards critical thinking
		Psychological well-being
		Psychological homeostasis
		Intrinsic motivation
		Logical reasoning skills
	Sociocultural factors Number of studies: (*n* = 13)	Access to resources
		Participatory and democratic culture
		Cultural diversity
		Cultural plasticity
	Technological factors Number of studies: (*n* = 17)	Dissemination of verified information
		Interaction with technological innovations
		Digital literacy
		Digital environment
		Technological capability
	Educational factors Number of studies: (*n* = 18)	Active pedagogy methodologies
		Problem-Based Learning
		Cooperative learning
		Active learning
		Explicit pedagogical approaches to integrating critical thinking

**Table 2:** Studies on physiological factors that influence critical thinking.

Physiological Factors (*n* = 7)				
No	Authors	Year	Title of Study	References
1	Ekerer et al.	2024	The effect of structured brain gym and brisk walking training on the executive functions of university students: a single-blinded randomised controlled trial.	(Ekerer et al. 2024)
2	López-Ruiz et al.	2021	Pensamiento crítico en estudiantes de educación superior: una revisión sistemática.	(López-Ruiz et al. 2021)
3	Mehta, K. J.	2022	Effect of sleep and mood on academic performance—at interface of physiology, psychology, and education.	(Mehta 2022)
4	Pereira et al.	2024	How sleep disorders can affect the intellectual development of college students.	(Pereira et al. 2024)
5	Ilyas et al.	2024	Am I over-sleeper? Oversleeping and cognitive functioning among university students.	(Sidra Ilyas et al. 2024)
6	Suárez et al.	2020	Influencia del estado nutricional, nivel de actividad física y condición física en el rendimiento académico de estudiantes universitarios.	(Suárez et al. 2020)
7	Wassenaar et al.	2020	A critical evaluation of systematic reviews assessing the effect of chronic physical activity on academic achievement, cognition and the brain in children and adolescents: a systematic review.	(Wassenaar et al. 2020)

**Table 3:** Studies on psychological factors related to the development of critical thinking.

Psychologycal Factor (*n* = 28)				
No	Authors	Year	Title of Study	References
1	Abanto-Ramirez et al.	2024	Disposition to critical thinking, anxiety due to COVID-19 and academic self-efficacy in university students.	(Abanto-Ramirez et al. 2024)
2	Amin et al.	2024	Debating as an Academic Tool: Fostering Holistic and Critical Thinking in Higher Education.	(Amin et al. 2024)
3	Arhip, G.	2022	The effects of mindfulness on critical thinking dispositions: Implications for mindful learning.	(Arhip 2022)
4	Aston	2024	‘Why is this hard, to have critical thinking?’ Exploring the factors affecting critical thinking with international higher education students.	(Aston 2024)
5	Berberena et al.	2021	Unveiling unconscious biases and stereotypes in students: The necessity of self-reflection in Higher Education.	(Berberena and Wirzberger 2021)
6	Chemagosi, M. J.	2024	Student Well-Being in Higher Education Institutions.	(Chemagosi 2024)
7	Crone et al.	2024	Critical Thinking in the Humanities: An Exploration of Student Perspectives.	(Crone and van Goch 2024)
8	Garcés-Suarez, E. F.	2024	El fortalecimiento del pensamiento crítico y las estrategias metacognitivas en estudiantes universitarios.	(Garcés-Suarez 2024)
9	Guamanga et al.	2025	Critical Thinking and Metacognition: Pathways to Empathy and Psychological Well-Being.	(Guamanga et al. 2025)
10	Huamán, G. R. L.	2022	Desarrollo del pensamiento crítico de los estudiantes a partir del aprendizaje basado en problemas: una revisión sistemática.	(Huamán 2022)
11	Huamán-Tapia et al.	2023	Critical thinking, generalized anxiety in satisfaction with studies: the mediating role of academic self-efficacy in medical students.	(Huamán-Tapia et al. 2023)
12	Hyseni Duraku et al.	2024	Overcoming mental health challenges in higher education: a narrative review.	(Hyseni Duraku et al. 2024)
13	Kazemian et al.	2021	Developing Metacognitive Writing Strategy to Enhance Writing Skills Viewed from Prospective Teachers’ Critical Thinking Skills.	(Kazemian et al. 2021)
14	Li, C. S.; Wan, R.	2022	Critical reading in higher education: A systematic review.	(Van et al. 2022)
15	Li, Y.	2024	Investigation on the Integration of Higher Education Logic Instruction and Cognitive Development.	(Li 2024)
16	Lugo et al.	2022	El pensamiento crítico en el contexto universitario: una vertiente del aprendizaje basado en problemas.	(Lugo et al. 2022)
17	Padmanabhan, S.	2024	Innovative Ways to Boost Well-Being Among Students in Higher Education.	(Padmanabhan 2024)
18	Pedorych, A. V.	2024	The study of logic as a condition for the development of critical thinking of higher education students.	(Pedorych 2024)
19	Rarita, M.	2023	An evaluation of the perceptions of socio-humanities students on the skills of critical thinking.	(Rarita 2023)
20	Rivas, S. F.; Saiz, C.; Ossa, C.	2022	Metacognitive strategies and development of critical thinking in higher education.	(Rivas et al. 2022)
21	Robison, M. K.; Brewer, G. A.	2022	Individual differences in working memory capacity, attention control, fluid intelligence, and pupillary measures of arousal.	(Robison and Brewer 2022)
22	Robison, M. K.; Garner, L. D.	2024	Pupillary correlates of individual differences in n-back task performance.	(Robison and Garner 2024)
23	Saiz, C.; Rivas, S. F.	2023	Critical thinking, formation, and change.	(Saiz and Rivas 2023)
24	Simonovic et al.	2022	The Impact of Attitudes, Beliefs, and Cognitive Reflection on the Development of Critical Thinking Skills in Online Students.	(Simonovic et al. 2022)
25	Smith, G.; Peloghitis, J.	2020	Critical Thinking and Debiasing: Experimentation in an Academic Writing Course.	(Smith and Peloghitis 2020)
26	Svetlana Antonova et al.	2021	Fostering Critical Thinking Skills in European and Asian Higher Education Institutions.	(Antonova et al. 2021)
27	Tianye Jia	2024	A Study of the Causes and Effects of Anxiety among Students in Higher Education.	(Jia 2024)
28	Zhao, C.; Vogel, E. K.	2025	Individual differences in working memory and attentional control continue to predict memory performance despite extensive learning.	(Zhao and Vogel 2025)

**Table 4:** Studies on sociocultural factors in strengthening critical thinking.

Sociocultural Factors (*n* = 13)				
No	Authors	Year	Title of Study	References
1	Abhishek Singla et al.	2024	Study on Analysing the Student Perspectives on Cultural and Social Diversity in Education.	(Singla et al. 2024)
2	Aboukhousa et al.	2024	Bridging Cultures for Global Environmental Sustainability: Empowering Change through Higher Education and Beyond.	(Aboukhousa et al. 2024)
3	Afzal et al.	2023	The role of teachers in fostering critical thinking skills at the university level.	(Afzal et al. 2023)
4	Darcie et al.	2024	Sustaining critical minds: How classroom learning cultures shape student thinking dispositions and practices.	(Darcie et al. 2024)
5	Escobar Domínguez, M.	2024	Práctica pedagógica del pensamiento crítico desde la psicología cultural.	(Escobar Domínguez 2024)
6	Koseni et al.	2025	The influence of the social environment on the development of critical thinking skills in young people: A study in the city of Durres, Albania.	(Koseni et al. 2025)
7	Liu, Y.; Pásztor, A.	2023	Survey on the influential demographic factors of Chinese undergraduate students’ critical thinking disposition: Evidence from plausible values.	(Liu and Pásztor 2023)
8	Mejía-Manzano et al.	2022	Embracing Thinking Diversity in Higher Education to Achieve a Lifelong Learning Culture.	(Mejía-Manzano et al. 2022)
9	Novoa Seminario et al.	2023	Estrategias para fortalecer el pensamiento crítico en estudiantes de educación superior. Un estudio de revisión.	(Novoa Seminario and Sandoval Rosas 2023)
10	Nuritdinov, N.	2024	Socio-Cultural Factors of the Effective use of Critical Thinking in the Development of Modern Legal Knowledge.	(Nuritdinov 2024)
11	Shukla, M.; Kumari, B.	2024	Exploring cultural and contextual factors impacting critical thinking skills among higher secondary students.	(Shukla and Kumari 2024)
12	Van Damme et al.	2023	Assessing and developing critical-thinking skills in higher education.	(Van Damme et al. 2023)
13	Walker, S.; LeBoeuf, S.	2022	Relationships in teaching for critical thinking dispositions and skills.	(Walker and LeBoeuf 2022)

**Table 5:** Studies on technological factors and their influence on critical thinking.

Technological Factors (*n* = 17)				
No	Authors	Year	Title of Study	References
1	Al-Zou’bi	2021	The impact of media and information literacy on acquiring the critical thinking skill by the educational faculty’s students.	(Al-Zou’bi 2021)
2	Bhuttah et al.	2024	Enhancing student critical thinking and learning outcomes through innovative pedagogical approaches in higher education: the mediating role of inclusive leadership.	(Bhuttah et al. 2024)
3	Fitriani & Prodjosantoso	2024	The Role of Technology in the Development of Critical Thinking: Systematic Literature Review.	(Fitriani and Prodjosantoso 2024)
4	Foote, K.; McMorran, C.	2024	Using technology to promote critical thinking.	(Foote and McMorran 2024)
5	Lopez-González et al.	2023	Educación mediática e informacional y pensamiento crítico: una revisión sistemática.	(Lopez-González et al. 2023)
6	Gonzalez-Mohino et al.	2023	Empowering critical thinking: The role of digital tools in citizen participation.	(Gonzalez-Mohino et al. 2023)
7	Jasmadi et al.	2024	Critical thinking: An analytical study on the impact of Artificial Intelligence (AI) usage on students at Al Washliyah Darussalam University, Banda Aceh.	(Jasmadi et al. 2024)
8	Kerruish, E.	2025	Critical thinking in higher education: Taking Stiegler’s counsel on the digital milieu.	(Kerruish 2025)
9	Mango-Quispe et al.	2024	Alfabetización mediática y pensamiento crítico en la formación inicial de docentes.	(Mango-Quispe et al. 2024)
10	Nguyen et al.	2024	Enhancing student engagement through artificial intelligence (AI): Understanding the basics, opportunities, and challenges.	(Nguyen et al. 2024)
11	Pedraja-Rejas et al.	2024	Mobile Learning and Its Effect on Learning Outcomes and Critical Thinking: A Systematic Review.	(Pedraja-Rejas et al. 2024)
12	Ravikumar et al.	2025	Advancing STEAM Education: Integrative Approaches, Challenges, and Future Directions.	(Ravikumar et al. 2025)
13	Tsopra et al.	2023	Putting undergraduate medical students in AI-CDSS designers’ shoes: An innovative teaching method to develop digital health critical thinking.	(Tsopra et al. 2023)
14	Valavičienė et al.	2020	Critical thinking embeddedness in higher education programmes.	(Valavičienė et al. 2020)
15	Van den Berg, G.; du Plessis, E.	2023	ChatGPT and generative AI: Possibilities for its contribution to lesson planning, critical thinking and openness in teacher education.	(Van den Berg and du Plessis 2023)
16	Vera, F.	2023	Integración de la Inteligencia Artificial en la Educación superior: Desafíos y oportunidades.	(Vera 2023)
17	Wang, Y.	2024	An interactive online educational environment to reduce anxiety, improve emotional well-being, and critical thinking for college students.	(Wang 2024)

**Table 6:** Studies on educational factors and pedagogical strategies for critical thinking.

Educational Factors (*n* = 18)				
No	Authors	Year	Title of Study	References
1	Abujreiban	2023	Impact of Using Some Active Learning Strategies in a Curricula and Teaching Methods Course on the Learning Motivation Level and the Development of Critical Thinking Skills Among the Students of the Faculty of Arts at Al-Zaytoonah University.	(Abujreiban 2023)
2	Alsaleh	2020	Teaching Critical Thinking Skills: Literature Review.	(Alsaleh 2020)
3	Campo et al.	2023	Methodologies for fostering critical thinking skills from university students’ points of view.	(Campo et al. 2023)
4	Cumandá et al.	2022	Desarrollo del pensamiento crítico a través de las tecnologías de la información y la comunicación en el nivel académico superior.	(Cumandá et al. 2022)
5	Dissen, A.	2023	A critical issue: assessing the critical thinking skills and dispositions of undergraduate health science students.	(Dissen 2023)
6	Garces et al.	2024	Habilidades lúdicas y su incidencia en el desarrollo del pensamiento crítico.	(Garces et al. 2024)
7	Huaire-Inacio et al.	2023	Desarrollo del pensamiento crítico en estudiantes universitarios.	(Huaire-Inacio et al. 2023)
8	Jones, J. A.; Irani, T. A.; Song, J.	2023	Critical Thinking in Nonprofit Management Education: Is an Explicit Emphasis Related to Greater Skill Development?	(Jones et al. 2023)
9	Marín Ube et al.	2025	Estrategias didácticas para fomentar el pensamiento crítico en el desarrollo de competencias comunicacionales: Una revisión sistemática de literatura con el Método SALSA.	(Marín Ube et al. 2025)
10	Mora-Ramírez, R. F.	2023	Uso de las paradojas como recursos didácticos que desarrollan el pensamiento crítico en los estudiantes.	(Mora-Ramírez 2023)
11	Moya Gómez, B. J.	2024	El juego como estrategia lúdica en el proceso enseñanza-aprendizaje.	(Moya Gómez 2024)
12	Pincay Parrales, M. L.	2022	Incidencia de la clase invertida en el desarrollo del pensamiento crítico.	(Pincay Parrales 2022)
13	Sánchez Gonzales et al.	2021	Uso de metodologías activas para el desarrollo de pensamiento crítico.	(Sánchez Gonzales and Nagamine Miyashiro 2021)
14	Song, H.; Cai, L.	2024	Interactive learning environment as a source of critical thinking skills for college students.	(Song and Cai 2024)
15	Sutherland, A.; Incera, S.	2021	Critical Reading: What Do Faculty Think Students Should Do?	(Sutherland and Incera 2021)
16	Thornhill-Miller et al.	2023	Creativity, critical thinking, communication, and collaboration: assessment, certification, and promotion of 21st century skills for the future of work and education.	(Thornhill-Miller et al. 2023)
17	Vélez et al.	2024	Desarrollo del pensamiento crítico mediante el uso de TIC en la formación de lenguaje y comunicación en la educación superior.	(Vélez et al. 2024)
18	Villarruel et al.	2021	Estrategias metodológicas y pensamiento crítico en la educación superior.	(Villarruel-Diaz and Portocarrero-Gutiérrez 2021)

## Data Availability

The original contributions presented in the study are included in the article, further inquiries can be directed to the corresponding author.
